# Chromatography-Olfactometry Study of the Aroma of Fino Sherry Wines

**DOI:** 10.1155/2010/626298

**Published:** 2010-06-24

**Authors:** L. Zea, L. Moyano, M. J. Ruiz, M. Medina

**Affiliations:** Department of Agricultural Chemistry, University of Cordoba, Campus de Rabanales, edificio Marie Curie, 14014 Cordoba, Spain

## Abstract

The aroma of Fino sherry wines produced by industrial biological aging for 0, 1.5, 2.5, 4.5, and 6 years in the Montilla-Moriles region (southern Spain) was studied by gas chromatography-olfactometry. The aroma sensations detected by 3 trained sniffers were classified according to their odor descriptors into 8 odorant series (fruity, empyreumatic, chemical, fatty, balsamic, vegetable, floral, and spicy), describing the aroma profile of the studied wines. The results showed 47 detected odors in the unaged wines, 50 in the 1.5-years-old wines and 59, 61 and 69 in the wines aged 2.5, 4.5, and 6 years, respectively. According to the frequency of the perceived aromas, the fruity and empyreumatic series were the most characteristic odorant series. By exception of chemical, floral and balsamic series without changes during aging of the wines, the remainder series increased their participation during the aging, mainly the fruity, empyreumatic, and fatty series.

## 1. Introduction

Sherry-type white wine called “Fino” is obtained through a long process (about 5-6 years) of biological aging under the action of so-called *flor* yeasts, which develop aerobically on the surface of wines containing 15–15.5% ethanol [1, 2]. Fino wine exhibits special sensory features including a pale yellow color, a slightly bitter flavor and a complex aroma. This last is developed during its biological aging, largely as a result of the action of *flor* yeasts as well as the contribution of volatile compounds extracted from the wood casks where the wine is aged. In addition, *flor* yeasts protect the wine from chemical browning, preserving its pale color throughout the aging period [3], and the contact of the wine with cask wood causes some of its components to be extracted, acquiring the wine its characteristic bouquet.

Essentially, the industrial aging method used in the area involves storing the wine in 500 L American oak barrels which are stacked in rows called *escalas *to construct a *criaderas and solera* system. The barrels in each *escala *contain the same wine at the same aging stage. The first *escala*, called *solera*, is closest to the ground and contains the oldest wine. A fraction from 1/3 to 1/4 of its contents is withdrawn 3-4 times a year for bottling. After each withdrawal, the barrels in the *solera *are replenished with wine from the second *escala*, also called first *criadera*, which in turn is replenished with wine from the third *escala *(second *criadera*) and so forth. The topmost *escala*, contains young wine from the year's vintage. 

The aroma profile of wine is rather complex. In fact, some analytical techniques such as GC–MS have allowed the identification of more than eight hundred volatile compounds. Many, however, are present at very low concentration levels [4]. In any case, instrumental techniques can detect chemicals, but are unable to identify those with a perceivable odor; in fact, only a small number of volatile compounds are odor active and contributors to wine aroma [5–8].

Gas chromatography–olfactometry (GC–O), also known as “sniffing”, is highly useful for establishing aroma profiles as it allows the discrimination of odor-active compounds in complex matrices [9–14]. In some cases, this discriminating ability is more than adequate; in most, however, the specific contribution of each individual odor compound to the overall aroma must be established. To this end, the GC–O has increasingly been used in combination with sophisticated olfactometric methods to estimate the sensory contribution of odor active compounds [15–17]. 

 In this work, changes in odor descriptors during biological aging of Fino sherry wines are studied with a view to estimating their different participation in the aroma profile and to establish the sequence of the odorant series which constitute the aroma fingerprint of these wines.

## 2. Material and Methods

Very pale sherry wines (fino type), selected by expert tasters as more representatives among the wines produced in 21 cellars from Montilla-Moriles region, were used. The wines were biologically aged for 0 (young wine), 1.5, 2.5, 4.5, and 6 years. This last wine is commercially considered as high-quality typical fino sherry wines. One sample for each aging year (or *escala*) was taken of each cellar. All wines of the same aging degree were mixed and immediately analyzed by triplicate.

The identification of each aroma compound was carried out by its retention time, coelution with a standard (purity >99%, Sigma Aldrich, Munich, Germany), and Mass Spectrometry (Hewlett-Packard 5972 MSD, CA, USA). The conditions of MS were scan mode and mass range from 39 to 300 amu. The chromatographic column, injector and oven temperatures, carrier gas and its flow were the same that those used for the sniff, being described below. 

Samples of 100 mL of wine were adjusted to pH 3.5, 150 *μ*g of 2-octanol was added as an internal standard and then extracted with 100 mL of freon-11 (Sigma-Aldrich Quimica, S.A., Madrid, Spain) in a continuous extractor for 24 hours (liquid-liquid extractor for use with solvents with higher density than sample). After concentration of the freon extracts to 0.2 mL in a micro-Kuderna-Danish concentrator, the GC-O analyses were carried out in a Hewlett-Packard-5890 series II gas chromatograph equipped with a sniffing port (Olfactory Detector, part. No. 093500, SGE-International, Australia) connected by a flow splitter to the column exit. The GC effluent was split 1 : 2 between the FID and the sniffing port. Humidified air was added in the sniffing port at 33 mL/min. Three microliters were injected into the chromatograph equipped with a HP-INNOWax column of 60 m × 0.32 mm × 0.25 *μ*m thickness (Agilent Technology, CA, USA). The oven temperature program was as follows: 5 min at 45°C, 1°C/min up to 185°C, and 30 min at 185°C. Injector and detector temperatures were 275°C and 300°C, respectively. The carrier gas was helium at 70 kPa and split 1 : 30. 

 Three trained judges, one woman and two men, selected for their ability to generate accurate terms as well as experienced in CG–O Sherry wines, performed the sniffing of the extracts. The panelists took turns for 15 minutes and again up to 140 minutes (total time of aromagram). Samples were sniffed three times, of same way previously mentioned, one session per day, and aroma descriptors and approximate times were recorded.

## 3. Results and Discussion

Table 1 shows the results of the GC–O analysis of the wine during the biological aging. Based on the high complexity of the odor profile, some compounds may be associated to two or three different notes [18, 19]. As in previous work, the most common odor descriptors were classified into aroma series in order to reduce the number of variables to be interpreted in establishing the aroma profile for the wines [1, 20].

The sniffers detected a total of 47, 50, 59, 61, and 69 different odors in wine from the fifth, fourth, third, second, and first cask row (*escala*), respectively. This suggests that aroma perceptions increase as the wine ages. The odors are grouped in three odorant zones (OZs) defined between two retention times (*R*_*t*_) for each and separated by a gap of at least 6 min with no odor perception. Thus, OZ_1_ spanned the aromagram region from 8.5 to 46.1 min, OZ_2_ from 57.8 to 64.6 min and OZ_3_ from 73.5 to 136.7 min. As can be seen in Table 1, the first and third zones were the most useful in sensory terms as they jointly accounted for more than 90% of total aroma notes detected in all *escalas*, whereas the second zone accounted for only about 10% of such notes.

Taking into account the citation frequency that an odor associated to a given aroma series was detected, it can be observed that OZ_1_ for fino wines was characterized largely by fruity series, followed by chemical and fatty series. Empyreumatic, vegetable, floral and balsamic odors were less frequently detected, and spicy notes were never detected, in this zone.

The fruity odors perceived (strawberry, apple, pineapple, peach, overripe melon, banana and almond) were all due to acetaldehyde and its derivative 1,1-diethoxyethane in addition to ethyl acetate, isobutanoate, butanoate, hexanoate, lactate, and octanoate, isoamyl acetate and an unidentified compound with *R*_*t*_ = 13.0 min. The number of fruity notes in OZ_1_ remained constant during the biological aging process; however, banana note was only detected in wine from the fifth and fourth *escalas* rows (0 and 1.5 years old, resp.); also, overripe melon notes were only detected in the third, second, and first *escalas* (2.5, 4.5 and 6 years old, resp.).

The descriptors associated to the fatty series detected in OZ_1_ included butter, cookie, and cheese. All were detected in every *escala* and due to the presence of 2,3-butanedione, methyl butanoate, acetoin and ethyl lactate throughout the aging process. This was also the case with the varnish, glue, alcohol, nail polish, vinous, and solvent odors, all in the chemical series, which were due to the presence of ethyl acetate, methyl butanoate, isobutanol, and isoamyl alcohols.

Empyreumatic odors were much less frequently detected than were those grouped in the previous series. This series included toasted notes in wine from the fifth to the second *escalas* due to an unidentified compound with *R*_*t*_ = 22 min. However, the first *escala* (6 years) exhibited a greater variety of empyreumatic notes including chocolate, coffee, toasted and burnt wood, the first three of which were due to unidentified compounds with retention times from 8.9 to 15 min and the last to furfural. The only vegetable (herbaceous) odor identified by the sniffers was detected below the third *escala* (2.5 years of aging) and due to octanal. Also, the only floral note was detected below the second *escala* and due to furfural. Finally, the sole balsamic (liqueur) odor detected was found in the youngest wine (fifth *escala*) and due to the presence of an unidentified compound with *R*_*t*_ = 11.5 min.

As noted earlier, the OZ_2_ was the smallest contributor to the aroma profile of the studied wines. In fact, it only encompassed odors in the fruity, empyreumatic and fatty series. Fruity notes were due to an unidentified compound with *R*_*t*_ = 57.8 min and *γ*-butyrolactone; none, however, was ascribed to a specific fruit. The number of fruity notes detected in this zone remained constant to the third *escala* (2.5 years) and then increased by effect of the contribution of the above-mentioned unidentified compound detected in the wines aged 4.5 years or more. The aroma notes associated to the empyreumatic (toasted, burnt) and fatty series (cheese, rancid) were due to *γ*-butyrolactone and butanoic acid, respectively, which remained practically constants throughout the aging period.

The odors in OZ_3_ were mostly related to the empyreumatic, spicy, fruity, and fatty series. The toasted, burnt, caramel, and coffee notes detected in wine from the fifth and fourth *escalas* (0 and 1.5 years old, respectively) were associated to two unidentified compounds with a retention time of 73.5 and 136.7 min, respectively, in addition to 4-ethylguaiacol and eugenol. Empyreumatic odors were stronger in the third and lower *escalas* by effect of the presence of toasted, burnt (*γ*-decalactone), smoked, and tobacco notes—the two lasts was due to an unidentified compound with *R*_*t*_ = 123.0 min.

The spicy notes detected in OZ_3_ included vanilla (*Z*-oak lactone), clove (4-ethylguaiacol), cinnamon (eugenol), and curry (sotolon). Clove and cinnamon odors were detected throughout the wine aging period, whereas vanilla odors were first detected in the fourth *escala* (1.5 years old wine) and curry odors in the third (2.5 years of aging)—hence spicy notes strengthened with aging of the wine.

The fruity aromas in OZ_3_ were described as peach, roast apple, and raisin, and due to two unidentified compounds with a retention time of 83.5 and 136.7 min, respectively, and also as unspecified fruity odors (*Z*-oak lactone and *γ*-decalactone). The fruity notes in OZ_3_ are increased substantially during aging of the wine. Thus, the youngest wine (fifth *escala*) only exhibited raisin notes, whereas that in the fourth *escala* additionally exhibited the fruity odor of *Z*-oak lactone; the third and second of *γ*-decalactone; and the first of peach and roast apple. The cheese, rancid, milky, and cookie notes in the fatty series were due to hexanoic and octanoic acids in addition to two unidentified compounds with a retention time of 119.0 and 128.5 min, respectively. Like fruity notes, these odors grew during aging of the wine, largely by effect of the gradual increase in milky notes.

The floral, chemical, vegetable, and balsamic notes were smaller contributors to OZ_3_; in fact, they only contributed a single note to each and their strength remained constant throughout the wine aging period. The detected odors included rose (phenethyl alcohol and its acetate), synthetic (unidentified compound with *R*_*t*_ = 73.5 min), cut hay (methionol), and medicinal (unidentified compound with *R*_*t*_ = 93.0 min).

**Figure 1:**
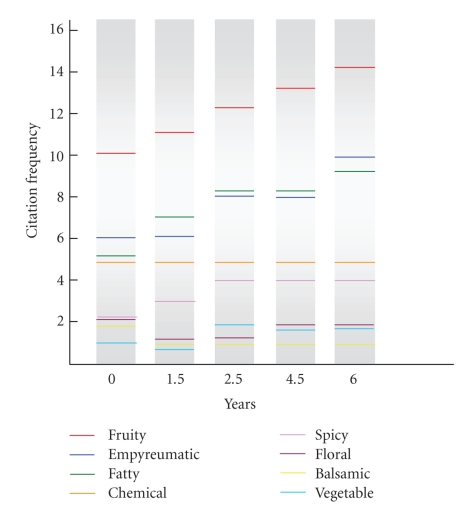
Aroma fingerprint for sherry type wine during the biological aging.

In order to show the total aroma notes of fino wine at various *escalas* of its biological aging, the odor descriptors for each one were arranged in increasing order of number of detections of the different aroma series (Figure 1). As can be seen, the highest citation frequency was the fruity series, which was that contributing the largest number of odor notes to the sensory profile of the wine throughout the aging process. Next came the empyreumatic and fatty series, followed by the chemical and spicy series, and, finally, the floral, balsamic and vegetable series. It should be noted that this sequence of series, which could constitute the aroma fingerprint of the wine, remained unchanged throughout the aging period; by exception, the fatty, vegetable and empyreumatic series exhibited slight changes in some *escalas* related to an increased contribution to the aroma of the wine. 

 In summary, the results of the GC–O analysis carried out during the aging of Fino wine revealed that all odorant series grew according to the citation frequency (particularly the fruity, empyreumatic and fatty ones) with time. By exception, the chemical, floral, and balsamic ones exhibited practically a constant contribution to the wine aroma throughout the aging process. Therefore, the compounds grouped in those series can be deemed the greatest contributors to the changes in aroma fingerprint during the biological aging of sherry wines.

## Figures and Tables

**Table 1:** Retention time, odor descriptors, and odorant series of the compounds detected by the sniffers grouped in three odorant zones (OZ).

	*R*_*t*_	Odor descriptors	Odorant series^(a)^	Compound	Aging years/*escala *				
					0/5th	1.5/4th	2.5/3th	4.5/2th	6/1th
OZ_1_	8.5	Pungent, fruity	Fr	Acetaldehyde	+	+	+	+	+
	8.9	Chocolate, coffee, toasted	E	n.i.	−	−	−	−	+
	9.4	Varnish, strawberry	Ch, Fr	Ethyl acetate	+	+	+	+	+
	9.6	Green apple	Fr	1,1-Diethoxyethane	+	+	+	+	+
	10.5	Apple, strawberry	Fr	Ethyl isobutanoate	+	+	+	+	+
	10.9	Butter, cookie	Fa	2,3-Butanedione	+	+	+	+	+
	11.1	Glue, fatty	Ch, Fa	Methyl butanoate	+	+	+	+	+
	11.5	Liqueur	B	n.i.	+	−	−	−	−
	12.2	Strawberry, pineapple, peach	Fr	Ethyl butanoate	+	+	+	+	+
	13.0	Overripe melon	Fr	n.i.	−	−	+	+	+
	13.2	Alcohol, nail polish	Ch	Isobutanol	+	+	+	+	+
	15.0	Toasted	E	n.i.	−	−	−	−	+
	15.3	Banana, strawberry	Fr	Isoamyl acetate	+	+	−	−	−
	19.9	Vinous, solvent	Ch	Isoamyl alcohols	+	+	+	+	+
	21.5	Fruity, almond	Fr	Ethyl hexanoate	+	+	+	+	+
	22.0	Toasted	E	n.i.	+	+	+	−	−
	27.0	Herbaceous	V	Octanal	−	−	+	+	+
	27.9	Butter, cheese	Fa	Acetoin	+	+	+	+	+
	34.5	Cookie, fruity	Fa, Fr	Ethyl lactate	+	+	+	+	+
	41.0	Fruity	Fr	Ethyl octanoate	+	+	+	+	+
	46.1	Floral, burnt wood	Fl, E	Furfural	−	−	−	+	+

OZ_2_	57.8	Fruity	Fr	n.i.	−	−	−	+	+
	63.0	Toasted, burnt, fruity	E, Fr	*γ*-Butyrolactone	+	+	+	+	+
	64.6	Cheese, rancid	Fa	Butanoic acid	−	+	+	+	+

OZ_3_	73.5	Toasted, synthetic	E, Ch	n.i.	+	+	+	+	+
	78.0	Cut hay	V	Methionol	+	+	+	+	+
	83.5	Peach, roast apple	Fr	n.i.	−	−	−	−	+
	84.6	Rose	Fl	Phenethyl acetate	+	−	−	−	−
	89.3	Cheese, rancid	Fa	Hexanoic acid	+	+	+	+	+
	93.0	Medicinal	B	n.i.	+	+	+	+	+
	99.4	Rose	Fl	Phenethyl alcohol	+	+	+	+	+
	103.0	Vanilla	S	*Z*-Oak lactone	−	+	+	+	+
	108.4	Toasted, clove	E, S	4-Ethylguaiacol	+	+	+	+	+
	113.7	Milky, rancid	Fa	Octanoic acid	−	−	+	+	+
	119.0	Milky, cookie	Fa	n.i.	−	+	+	+	+
	121.0	Toasted, burnt, fruity	E, Fr	*γ*-Decalactone	−	−	+	+	+
	123.0	Smoked, tobacco	E	n.i.	−	−	+	+	+
	125.6	Burnt, cinnamon	E, S	Eugenol	+	+	+	+	+
	128.5	Milky, cookie	Fa	n.i.	−	−	−	−	+
	131.0	Curry, tobacco	S	Sotolon	−	−	+	+	+
	136.7	Caramel, coffee, raisin	E, Fr	n.i.	+	+	+	+	+

^(a)^Fr: fruity; E: empyreumatic; Ch: chemical; Fa: fatty; B: balsamic; V: vegetable; Fl: floral; S: spicy

n.i.: not identified.

The “+” indicates that sensation was detected by at least two sniffers and the “−” when the odor was detected only by one or none sniffers.
